# Factors associated with a willingness to accept latent tuberculosis infection treatment among non-U.S.–born individuals: a situational choice experiment

**DOI:** 10.64898/2026.09.09.26362665

**Published:** 2026-09-12

**Authors:** Sutina Chou, Hélène E. Aschmann, Amy S. Tang, Meagan Lee, Zinnia Dong, Kit Lui, Yuqian Ouyang, Gina Chen, Katya L. Salcedo, Matthew Murrill, Mehabuba Rahman, Jennifer Flood, Andrew D. Kerkhoff, Priya B. Shete, Tracy Kuo Lin, the Tuberculosis Epidemiologic Studies Consortium

## Abstract

**Introduction:**

Despite efforts to eliminate tuberculosis (TB) in the United States (U.S.), many individuals diagnosed with latent TB infection (LTBI) who are at increased risk of progressing to TB disease do not initiate TB preventive treatment.

**Methods:**

Among adults receiving primary care in a federally qualified community health center in the San Francisco Bay Area, California, we conducted a cross-sectional online survey using a situational choice experiment to assess the circumstances in which individuals at risk for TB based on nativity would accept LTBI treatment. In 10 randomized choice tasks, participants answered whether or not they would accept LTBI treatment. In situations where LTBI treatment was accepted, participants were asked a follow-up question about whether they would change their response knowing that reinfection is still possible with LTBI treatment. Attributes assessed included risk level of progression to TB, side effects, changes to co-medication, cost, number of clinic visits, blood draws, and reinfection risk. We conducted a mixed-effects logistic regression to estimate factors associated with treatment acceptance and predicted acceptance across scenarios.

**Results:**

Of 852 individuals who consented, 458 (53.8%) had complete responses and were included in the analysis. LTBI treatment was accepted in 69.4% of choice scenarios (3180/4580); among scenarios where treatment was initially accepted, 91.5% of scenarios (2911/3180) remained accepted when reinfection possibility was introduced. Compared to situations with low risk of TB progression, high TB progression risk increased the acceptance of LTBI treatment (adjusted odds ratio aOR 2.55, 95% Confidence Interval (CI) = 2.03–3.19). Compared with fatigue as a referent, risk of any liver injury/inflammation (aOR 0.29, 95% CI = 0.22–0.38), gastrointestinal side effects (aOR 0.53, 95% CI 0.41, 0.69), rash (aOR 0.62, 95% CI 0.48-0.80) and out-of-pocket cost of $100 (aOR 0.32, 95% CI = 0.26–0.41 compared to $0) were associated with lower acceptance of LTBI treatment.

**Conclusions:**

The decision to accept LTBI treatment was sensitive to treatment characteristics, suggesting that shared decision-making should be broadly promoted among individuals diagnosed with or at risk of LTBI, particularly in diverse primary care settings serving individuals born in high TB burden countries, with attention to individual concerns such as cost and side effects to improve LTBI treatment uptake.

## Full Text Availability

The license terms selected by the author(s) for this preprint version do not permit archiving in PMC. The full text is available from the preprint server.

